# Viral DNA integration and methylation of human papillomavirus type 16 in high-grade oral epithelial dysplasia and head and neck squamous cell carcinoma

**DOI:** 10.18632/oncotarget.25754

**Published:** 2018-07-13

**Authors:** Sujita Khanal, Brian S. Shumway, Maryam Zahin, Rebecca A. Redman, John D. Strickley, Patrick J. Trainor, Shesh N. Rai, Shin-je Ghim, Alfred Bennett Jenson, Joongho Joh

**Affiliations:** ^1^ Division of Human Biology, Fred Hutchinson Cancer Research Center, Seattle, WA, USA; ^2^ Department of Surgical and Hospital Dentistry, University of Louisville School of Dentistry, Louisville, KY, USA; ^3^ James Graham Brown Cancer Center, University of Louisville, Louisville, KY, USA; ^4^ Department of Medicine, School of Medicine, University of Louisville, Louisville, KY, USA; ^5^ Center for Predictive Medicine, University of Louisville, Louisville, KY, USA

**Keywords:** human papillomavirus (HPV), HPV integration, HPV methylation, high-grade oral epithelial dysplasia (hgOED), head and neck cancer

## Abstract

This study evaluated the integration and methlyation of human papillomavirus type 16 (HPV16) in head and neck squamous cell carcinoma (HNSCC) and its oral precursor, high-grade oral epithelial dysplasia (hgOED). Archival samples of HPV16-positive hgOED (*N* = 19) and HNSCC (*N* = 15) were evaluated, along with three HNSCC (UMSCC-1, -47 and -104) and two cervical cancer (SiHa and CaSki) cell lines. HgOED cases were stratified into three groups with increasing degrees of cytologic changes (mitosis, karyorrhexis and apoptosis). The viral load was higher and the E2/E6 ratio lower (indicating a greater tendency toward viral integration) in group 3 than in groups 1 or 2 (*p* = 0.002, 0.03). Methylation was not observed in hgOED cases and occurred variably in only three HNSCC cases (26.67%, 60.0% and 93.3%). In HNSCC cell lines, lower E7 expression correlated with higher levels of methylation. HgOED with increased cytologic change, now termed HPV-associated oral epithelial dysplasia (HPV-OED), exhibited an increased viral load and a tendency toward DNA integration, suggesting a potentially increased risk for malignant transformation. More detailed characterization and clinical follow-up of HPV-OED patients is needed to determine whether HPV-OED is a true precursor to HPV-associated HNSCC and to clarify the involvement of HPV in HNSCC carcinogenesis.

## INTRODUCTION

Head and neck squamous cell carcinoma (HNSCC) originates from multiple anatomic sites, including the oral cavity (oral squamous cell carcinoma, OSCC) and the oropharynx (oropharyngeal squamous cell carcinoma, OPC). While excessive tobacco and alcohol exposure are proven risk factors contributing to many cases of HNSCC, in other cases (particularly OPC), tumorigenesis is driven by infection with one of the high-risk types of human papillomavirus (HRHPV). Of note, the response to treatment and survival are better in HPV-associated cancers than in those that lack the virus, independent of the treatment strategy [1, 2]. While HPV is strongly associated with OPC, the relationship of HPV to OSCC and its premalignant precursor (high-grade oral epithelial dysplasia, hgOED) is not well understood. Our group [3] and others [4, 5] have recently reported that HRHPV is strongly associated with a certain histologic subset of hgOED, which is now designated as HPV-associated oral epithelial dysplasia (HPV-OED).

Our recent study [3] better established the histologic criteria for HPV-OED. We stratified hgOED cases based on a cytologic score determined by increasing numbers of mitotic, karyorrhectic and apoptotic cells per high-power field (hpf) (group 1 [0.0 to <1.7]; group 2 [1.7 to <5.3]; group 3 [≥5.3]). The odds of detecting HPV by PCR were 5.83 times greater in group 3 than in groups 1 and 2 combined. HRHPV was detected in 83.3% (10/12) of group 3 cases vs. 42.3% (11/26) of group 1 and 2 cases, and HPV16 was the type most often observed (90.5%) for all HRHPV-positive cases. Group 3 lesions were also more likely to display diffuse p16 expression by immunohistochemistry, and the combination of the cytologic score and p16 expression was a specific predictor for HRHPV. We did not observe a difference in the clinical progression of the disease among the groups, but our long-term follow-up was limited. We concluded that cases of hgOED with greater cytologic change (group 3) are strongly associated with HPV, warranting the designation of HPV-OED. However, because HPV16 was still identified in 42.3% of the lesions in groups 1 and 2, additional molecular study of these groups is needed to assess whether these differences determine the influence of HPV on the progression from dysplasia to malignancy.

Viral DNA integration and methylation are considered to be two major regulatory mechanisms for malignant transformation [6–9]. Persistent expression of HPV oncogenes (i.e., *E6* and *E7*) is necessary for cancer development, and the expression of these genes is mainly regulated by viral protein E2. When this transcription factor binds to the early promoter p97 at specific E2-binding sites (E2BSs) located within the HPV long control region (LCR), *E6* and *E7* expression are reduced [10, 11]. Overexpression of *E6* and *E7* can be caused either by disruption of the *E2* gene via HPV genome integration or by inhibition of E2 protein binding to the LCR via HPV methylation [12].

Integration of HRHPV into the host genome has been well characterized in cervical cancer [13–18] and is thought to be a key factor in the development of malignancy [7]. In HNSCC, the data on integration (focused on HPV16) have been inconsistent and incomplete, so the relevance of integration to head and neck carcinogenesis has been unclear [19]. Thus, we investigated the involvement of viral integration in HNSCC by examining three HNSCC cell lines that are associated with HPV16 and have demonstrated integration [20, 21]. The integration rates in HPV16-positive clinical samples of HNSCC have varied significantly, from 0% [22] to 100% [23, 24], but most studies have reported a range of 40–80% [25]. Some of this variation may be due to the various methods of detection [25].

In the oropharyngeal area specifically, OPC has exhibited higher rates of HPV integration [19, 22–24, 26–32] than OSCC [28, 29, 31, 33, 34], but far fewer OSCC cases have been evaluated. Only two studies have investigated HPV16 integration in head and neck epithelial dysplasia; both of them evaluated dysplastic marginal tissue adjacent to existing OPC, and reported high integration rates [24, 35]. A major reason for this lack of data is the subtle presentation of OPC, which often arises in the tonsillar crypts without a clinically visible surface premalignant lesion [24, 25, 35]. While OSCC is commonly preceded by clinically visible white and/or red alterations, biologically relevant HRHPV infections (i.e., E6, E7 expression) have only been observed in approximately 6% of cases [36], making the acquisition of HPV-infected lesions a rarity. However, in our recent study [3], we identified a histologic subset of hgOED that was strongly associated with HPV16, and specimens from the same cohort were used in the present study to assess integration in oral premalignancies.

DNA methylation is another potential factor in the malignant transformation of the HPV-infected epithelium. Methylation of the HPV genome, which contains 15 CpG sites in the LCR, has been suggested as a biomarker for cervical cancer progression [8, 9]. In the context of cervical cancer, methylation of HPV DNA prevents E2 from binding, thus releasing transcriptional repression and upregulating viral oncoproteins. It is not clear whether viral genome methylation is associated with malignancy in HNSCC in the same way that it is in cervical cancer, but an association has recently been suggested [37]. Paradoxically, one large study reported that the viral LCR was hypomethylated in oropharyngeal cancers, making it clear that more work is needed [38]. To our knowledge, apart from studies of host genome methylation, the HPV DNA methylation pattern in premalignant hgOED lesions has not been evaluated. Thus, we investigated the involvement of HPV DNA methylation in the malignant transformation of HNSCC.

To address the aforementioned research questions, we analyzed the physical state of HPV DNA (i.e., integrated or episomal) and the presence of potential DNA methylation sites in the HPV epigenome in patients with hgOED and HNSCC. Our findings have elucidated some of the complex mechanisms involved in HPV-induced HNSCC carcinogenesis.

## RESULTS

### Viral load in HPV-positive cancer cell lines

HPV16 DNA was detected in the UMSCC-47 and -104 cell lines, but not in UMSCC-1. Relative to the copy number in SiHa cells, 325 copies of HPV16 DNA were found in CaSki cells (Table 1); CaSki cells are known from previous studies [39] to have variable HPV DNA copy numbers (60–600). For both of the HPV16-positive HNSCC cell lines (UMSCC-47 and UMSCC-104), two viral copies were found (Table 1).

**Table 1:** Characteristics of cervical and head and neck cancer cell lines

Cell lines	HPV infection	^a^Viral load	DNA copy number		Full E2 DNA PCR	^c^mRNA expression		
			^b^E2/E6	Integration status		E6/E7 ^d^(q6/q7 = ratio)	p16^INK4a^	EGFR
SiHa	HPV16	2	0/0.1 = 0.0	integrated	no	^e^N.T	N.T	N.T
CaSki	HPV16	325	2.6/21.8 = 0.12	^f^mixed	yes	N.T	N.T	N.T
UMSCC-1	uninfected	^g^N.A	N.A	N.A	N.A	N.A	0.46	0.68
UMSCC-47	HPV16	2	0/0.08 = 0.0	integrated	yes	2.0/0.08 = 24.0	2.5	0.71
UMSCC-104	HPV16	2	0.003/0.068 = 0.045	mixed	yes	1.25/12.54 = 0.09	1.84	0.93

^a^Viral copies per cell.

^b^Ratio of *E2* (picograms, pg) to *E6* (pg) as determined from the standard curves of the respective genes by a previously. described quantitative real-time PCR assay [44]

^c^mRNA expression: By the ΔΔCt method (The values are expressed in percentage of 2^-ΔCt^).

^d^q6/q7 = quantification (pg) of *E6* mRNA/quantification (pg) of *E7* mRNA

^e^N.T: not tested.

^f^Mixed: This sample contains both episomal and integrated forms of HPV.

^g^N.A: not applicable.

### HPV16 DNA integration frequency into cell line host genomic DNA

**Figure 1:**
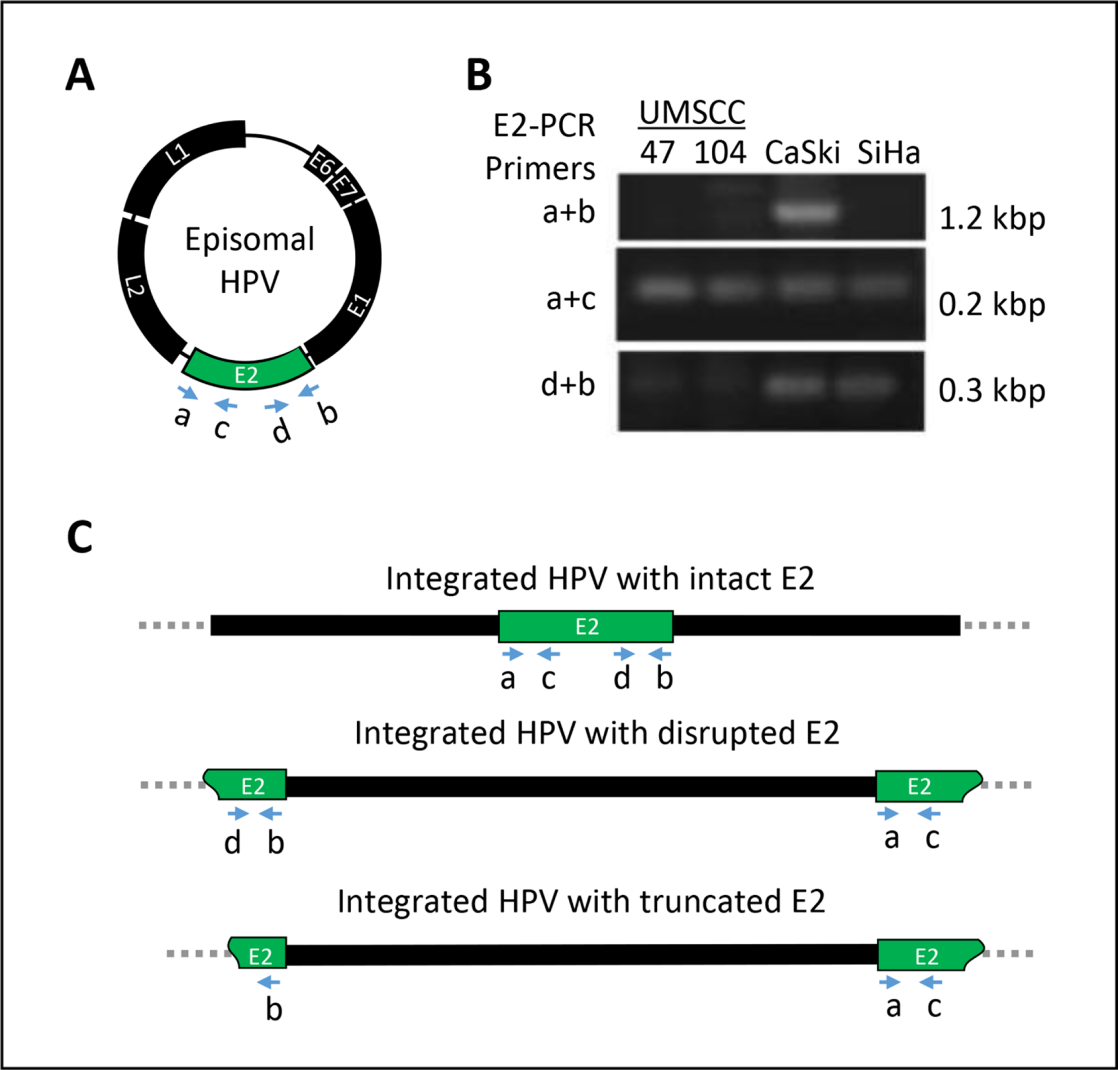
Determination of E2 gene integrity (**A**) by means of HPV16 *E2* primers (16E2 a, b, c and d, Supplementary Table 1) which detect the intact or disrupted E2 gene. (**B**) Agarose gel image displaying the full-length *E2* in CaSki cells and disrupted *E2* sequences in SiHa, UMSCC-47 and UMSCC-104 cells. (**C**) Schematic representation of three different ways that HPV DNA can integrate into the host chromosome.

In terms of integration status, SiHa cells exhibited an *E2/E6* ratio of 0.0, because *E2* was not detected, while 0.1 pg of *E6* was detected per 20 ng of total DNA (Table 1). This result indicated that both copies of HPV16 DNA in the SiHa cells were likely integrated into the chromosomal DNA via disrupted *E2* sites, as reported previously [39]. HPV DNA integration after *E2* disruption was further confirmed through the amplification of the *E2* open reading frame with nested PCR primers (Figure 1A). While small 3′- or 5′- *E2* fragments were amplified, no full-length *E2* was detected in SiHa cells (Table 1). CaSki cells exhibited an *E2/E6* ratio of 0.12, indicating that the majority of the HPV DNA in this cell line was mixed (Table 1). When calculated relative to that of the SiHa cell line, the *E2/E6* ratio of CaSki cells indicated that 236 integrated and 39 episomal HPV16 DNA copies were present per cell. Accordingly, both full-length and small fragments of the *E2* open reading frame were amplified in CaSki cells (Figure 1B), confirming the presence of mixed forms of intact and disrupted *E2* DNA.

HPV DNA integration was also determined through the evaluation of the *E2/E6* ratio in the UMSCC-47 and -104 cell lines. The *E2/E6* ratios in these cells were 0.0 and 0.045, respectively (Table 1), suggesting that both cell lines contained integrated HPV predominantly in disrupted *E2* sites. The copy number relative to that of the SiHa cell line indicated that both copies of HPV16 DNA were integrated in the UMSCC-47 cell line. In UMSCC-104 cells, a small number of episomal DNA copies (0.9 × 10^–4^ copies) existed when the two copies of DNA were integrated into the host genome. Full-length *E2* was not detected in either cell line by PCR (Figure 1B), confirming that the episomal form was either not detectable or not present in these cell lines.

### Transcriptional characterization related to integrated HPV DNA

**Figure 2:**
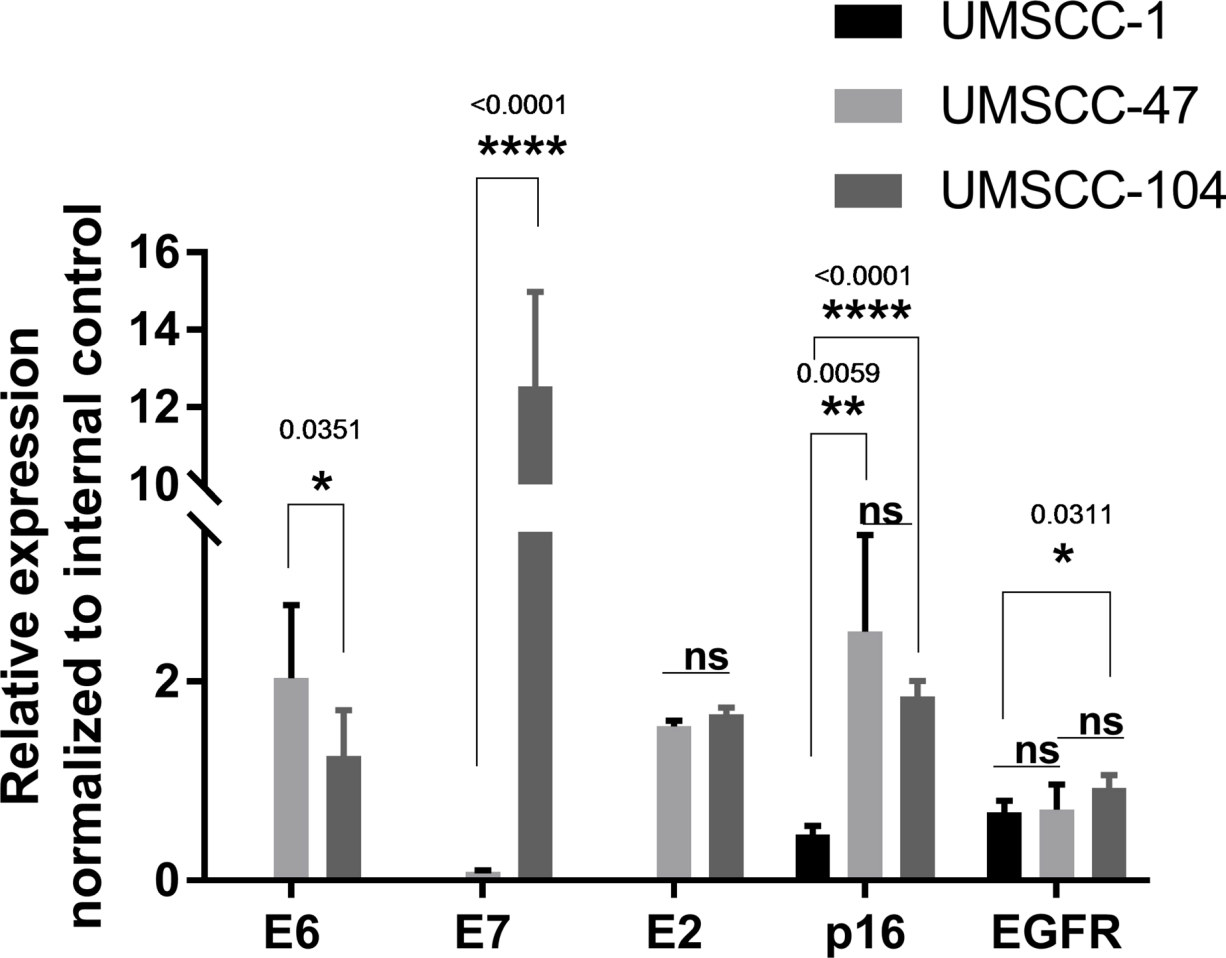
Relative expression of *E6*, *E7*, *E2*, *p16* and *EGFR* in UMSCC-1, UMSCC-47 and UMSCC-104 head and neck cancer cell lines *P*-values < 0.05 were considered significant. Statistical significance levels at ^*^p < 0.05, ^**^p < 0.01 and ^****^p < 0.0001 are indicated. ns represents non-significant. Statistical analyses were performed with Student's *t*-test (unpaired two-tailed) in GraphPad Prism. E2 primers were designed upstream of the E2 breakpoint.

Because the disruption of the *E2* gene upon HPV DNA integration causes robust and constitutive expression of the viral *E6* and *E7* oncogenes in cervical cancer [13–18], the expression profiles of the *E2*, *E6* and *E7* genes were tested in HNSCC cell lines (Table 1). Quantitative reverse-transcription (qRT)-PCR assays demonstrated that *E6* and *E7* mRNA levels varied considerably between the two studied HPV-positive HNSCC cell lines (Figure 2). *E7* expression was 150-fold higher in UMSCC-104 than in UMSCC-47 cells (*p* < 0.001); however, *E6* expression was approximately two-fold higher in the latter than in the former (*p* < 0.0351). *E2* mRNA expression was analyzed with primers designed to anneal upstream of the frequent *E2* breakpoint, and the two cell lines exhibited similar *E2* levels.

The levels of p16^INK4a^ and EGFR have also been associated with HNSCC [40, 41], so the expression of these genes was examined in HNSCC cell lines by qRT-PCR (Table 1). The expression of *p16^INK4a^* was similar in the HPV-positive UMSCC-47 and UMSCC-104 cell lines, and was significantly higher in these cells than in the HPV-negative UMSCC-1 cell line (by 5.4- and 4.0-fold, respectively). *EGFR* expression was 1.4-fold higher in UMSCC-104 than in UMSCC-1 cells, but did not differ significantly different between UMSCC-47 and UMSCC-1 cells or between UMSCC-104 and UMSCC-47 cells (Figure 2).

### Viral load and HPV16 DNA integration rates

**Figure 3:**
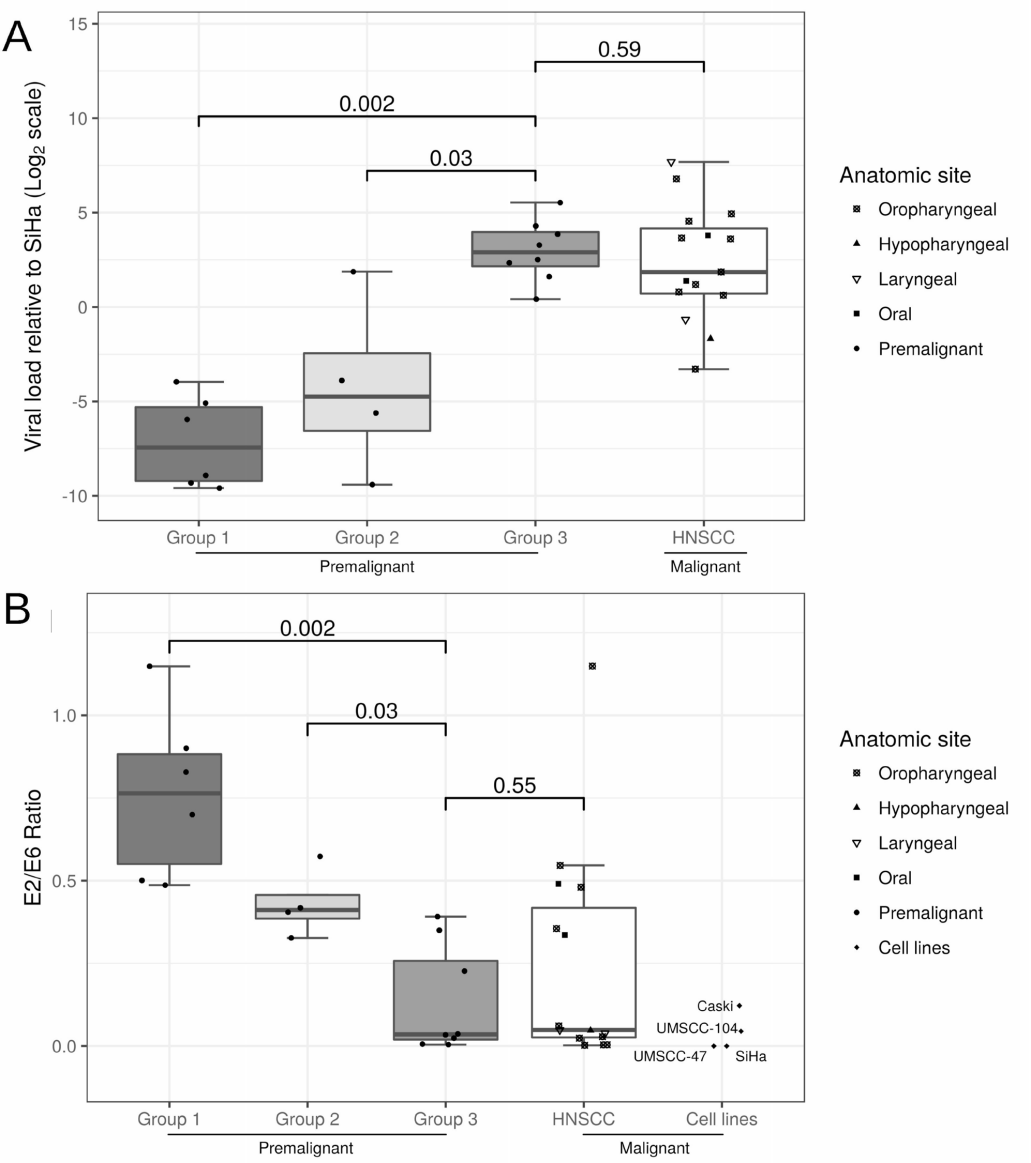
**(A)** Box plot of the viral load in each sample normalized to the level in SiHa cells and (**B**) box plot of the *E2/E6* ratio, indicative of the degree of viral DNA integration. In both analyses, premalignant cases (group 1: *N* = 6, group 2: *N* = 4 and group 3: *N* = 8) were compared with malignant samples (HNSCC), while the four cancer cell lines were added to the second plot. Pairwise Wilcoxon rank-sum tests were conducted, and multiplicity-adjusted *p*-values (Holm's method) are presented in the figure.

In our previous study of hgOED cases stratified into three groups according to the number of karyorrhetic, apoptotic and mitotic elements per hpf, increases in these cytologic alterations correlated strongly with the presence of HRHPV (particularly HPV16) and with higher p16 expression [3]. In the current study, HPV16-positive hgOED cases within each of these three cytologic groups (group 1 [*N* = 6], group 2 [*N* = 4], group 3 [*N* = 9]) were evaluated for between-group differences in HPV copy number and integration status. Of note, one case in group 3 was excluded from the analysis due to repeatedly aberrant results. Significantly higher viral copy numbers were observed in group 3 than in group 1 (*p* < 0.001) or group 2 (*p* = 0.016, Mann-Whitney test, Figure 3A). The HPV copy number in group 3 lesions was found to range from 1–46 copies. In contrast, all samples in groups 1 and 2 had relative viral loads of less than 0.1, with one exception of 3.6 copies in group 2 (Table 2).

**Table 2:** Viral loads and *E2/E6* ratios in HPV16-positive hgOED and HNSCC

Epithelial disease category		Histologic group^a^	Viral load^b^, median [Q1, Q3] *p*-value vs. G3	E2/E6 ratio^c^, median [Q1, Q3] *p*-value vs. G3
Premalignant Lesions (hgOED)		G1	0.009 [0.002, 0.026] *p* = 0.002	0.76 [0.55, 0.88] *p* = 0.002
		G2	0.04 [0.02, 0.97] *p* = 0.03	0.41 [0.39, 0.46] *p* = 0.03
		G3	7.75 [4.54, 15.81] N.A.	0.04 [0.02, 0.26] N.A.
Malignant lesions (HNSCC)	All HNSCC	N.A.	3.61 [1.64, 18.54] *p* = 0.59	0.05 [0.03, 0.42] *p* = 0.55
	Oropharyngeal Only	N.A.	7.93 [1.87, 20.54]^d^ *p* = 0.83^e^	0.04 [0.01, 0.45]^d^ *p* = 0.97^e^

*Abbreviations*: G1: group 1; G2: group 2; G3: group 3.

^a^Histologic groups (G1, G2, G3) as reported by Khanal *et al*. [3].

^b^Viral load normalized to that of SiHa cells.

^c^*E2/E6* ratio used to determine DNA integration status.

^d^Summary statistics for the malignant HNSCC group after the removal of non-oropharyngeal samples (*N* = 10).

^e^Statistical significance of oropharyngeal samples only: The *p*-value for “All HNSCC” represents the statistical significance of the Wilcoxon rank-sum test with all malignant samples (*N* = 15) included, while the *p*-value for “Oropharyngeal Only” represents the statistical significance after the removal of non-oropharyngeal samples (*N* = 10) (sensitivity analysis).

HPV integration status differed significantly among the hgOED groups (*p* = 0.002 for group 1 vs. group 3, Table 2, Figure 3B). The *E2/E6* ratio results indicated a strong tendency toward fully or predominantly integrated virus forms in group 3 (0.04) vs. mixed forms and a trend toward episomal forms in group 2 (0.41) and group 1 (0.76). In the 16 cases of HNSCC, variable viral loads (0.1–205 copies/cell) were found when the copy numbers were decided relative to SiHa (Table 2), and the median viral load did not differ significantly from that of group 3 hgOED (*p* = 0.59). HPV was integrated or predominantly integrated in 75% of the HNSCC cases, and the *E2/E6* ratio did not differ significantly from that of group 3 hgOED (*p* = 0.55). In a sensitivity analysis comparing the *E2/E6* ratios of only the OPC cases (*N* = 10) to those of the group 3 hgOED cases, there was still no significant difference (*p* = 0.97).

### HPV LCR methylation status

All the CpG target sites of the cellular methyltransferase were identified within the HPV16 LCR, which contains the promoter and various transcription factor binding sites. CpGs were found in 15 sites each in SiHa, CaSki and UMSCC-104 cells and at 13 locations in UMSCC-47 cells, due to two reported point mutations in the latter (nucleotide 7434 CG>CA and nucleotide 31 CG>TG) [42]. The binding sequences that contain CpGs are indicated in Table 3, along with the nucleotide location of each CpG site. A variety of promoter methylation patterns were observed in the four cancer cell lines. We observed evidence of hypermethylation in CaSki and UMSCC-47 cells (93.33% and 76.92% of the available CpGs, respectively). The two cells lines shared one unmethylated site (nucleotide 7862 within E2BS2), and UMSCC-47 had additional unmethylated CpGs at nucleotides 7676 and 7682. In contrast, all 15 CpG sites within the LCR were unmethylated in SiHa and UMSCC-104 cells.

**Table 3:** Methylation of CpG sites in cervical and OSCC cell lines and hgOED and HNSCC specimens

	5′-LCR				Enhancer					Promoter						
HPV genome	YY1		E2BS1		NF1	NF1	YY1	AP1	Tef	E2BS2	SP1	E2BS3		E2BS4		TATA
**CpG site^#^**	1	2	3	4	5	6	7	8	9	10	11	12	13	14	15	p97
**NT** **Sample^a^**	7428	7434	7455	7461	7535	7553	7676	7682	7694	7862	31	37	43	52	58	**% Methylation**
SiHa	U	U	U	U	U	U	U	U	U	U	U	U	U	U	U	0
CaSki	M	M	M	M	M	M	M	M	M	U	M	M	M	M	M	93.33
UMSCC-47b	M	Mut	M	M	M	M	U	U	M	U	Mut	M	M	M	M	76.92
UMSCC-104	U	U	U	U	U	U	U	U	U	U	U	U	U	U	U	0
HNSCC 2	M	M	M	M	U	U	U	U	U	U	M	M	M	M	M	60.0
HNSCC 7	M	M	M	M	M	M	M	M	M	U	M	M	M	M	M	93.33
HNSCC 12	U	U	U	U	U	M	U	U	U	U	M	U	U	M	M	26.67

Abbreviations: YY1, NF1, AP1, Tef, SP1: transcription factor binding sites; TATA: Tata box sequence; ^#^number; NT: nucleotide position; p97: Early promoter of HPV at 97 NT; U: unmethylated; M: Methylated; Mut: mutated.

^a^The remaining 12 HNSCC and all 18 premalignant hgOED specimens were unmethylated at all 15 CpG sites

^b^UMSCC-47 only has 13 CpG sites due to two known point mutations.

Methylation analysis revealed that all the CpG sites were unmethylated in all the samples in all three hgOED groups. Similarly, except for three samples exhibiting methylation (93.33%, 60% and 26.67% methylation respectively, Table 3), the HNSCC specimens were predominantly unmethylated within the LCR. Thus, there was an overall pattern of hypomethylation of the HPV epigenome in the patient samples.

## DISCUSSION

While viral integration and methylation are key processes in HPV-associated cervical cancer [7, 13, 14, 43], their involvement in head and neck carcinogenesis is not well understood. We used human hgOED and HNSCC samples and HNSCC cell lines to explore patterns of HPV DNA integration and methylation that could contribute to tumor development.

It is well documented [44] that SiHa cells have two copies of HPV16 DNA integrated into the host genome, as indicated by disrupted *E2* sequences. Therefore, we compared the integration patterns of other cell lines and the hgOED samples with that of SiHa cells (Table 1 and Figure 1). Our data indicated that CaSki cells contained a mixture of integrated and episomal HPV16 DNA (Table 1). The HPV-positive HNSCC cell lines, UMSCC-47 and UMSCC-104, exhibited similar copy numbers of HPV16 DNA, and possessed integrated or both episomal and integrated (i.e., mixed) forms of HPV, respectively (Table 1). We found a very weak PCR band of full-sized *E2* DNA in UMSCC-104 cells (Table 1 and Figure 1B), indicating the presence of episomal viral DNA. These results are consistent with data from Akagi *et al*. [21], but conflict with the results of Olthof *et al*., who reported the presence of solely episomal HPV in UMSCC-104 cells [20]. This discrepancy may be attributed to Olthof's use of a set of primers that did not span the full length of *E2*, and to the unique integration breakpoint of this cell line (Figure 1C), as reported by Akagi *et al*. [21]. Additionally, we found evidence of *E2* mRNA expression in both HNSCC cell lines (Figure 2), in contrast to a previous report that the *E2* gene was not intact and *E2* mRNA was not detectable in UMSCC-104 cells [45].

Since both of the HPV-positive HNSCC cell lines in this study contained integrated HPV DNA, it was not possible to make definitive conclusions about the relationship between viral oncogene expression and integration status. However, we did find significant differences in *E2*, *E6* and *E7* expression between UMSCC-47 (integrated DNA) and UMSCC-104 (mixed DNA with predominantly integrated HPV) cells that were worthy of exploration (Figure 3). While *E2* mRNA expression was slightly higher in UMSCC-104 cells than in UMSCC-47 cells, the difference was not statistically significant. This lack of difference was due to a limitation of the assay, in that the primers bind upstream of the integration breakpoint in *E2*, enabling the detection of functional and truncated *E2* mRNA in UMSCC-47 cells.

*E6* oncogene mRNA expression was significantly greater (*p* < 0.0351) in UMSCC-47 than in UMSCC-104 cells (Figure 2). We interpret these results to indicate that the E2 protein transcribed from integrated HPV in UMSCC-47 cells was truncated and nonfunctional, and thus was unable to perform its function as a transcriptional repressor of *E6*. In contrast, the E2 protein from the episomal HPV in UMSCC-104 was functional and expressed at a slightly higher level, and thus was able to repress *E6* expression. However, in the case of the *E7* oncogene, a large difference (*p* < 0.001) in mRNA expression was found. This expression profile was unexpectedly the opposite of that of *E6*, with higher expression in the UMSCC-104 cell line. This finding suggests that a mechanism unrelated to transcriptional repression by E2, such as epigenetic regulation (to be discussed shortly) may be related to HPV *E7* oncogene expression.

The overexpression of p16^INK4a^ has been observed in HPV-positive cancers [40, 41]. Consistently, we found that *p16^INK4a^* expression was significantly higher in the HPV-positive HNSCC cell lines than in the HPV-negative UMSCC-1 cell line. The E7 protein is known to upregulate p16^INK4a^ by inactivating pRB [46], but we could not find any relationship between *E7* and *p16^INK4a^* expression in the cell lines we tested. Another marker used clinically is EGFR, which is frequently overexpressed in HNSCC independent of HPV etiology [47, 48] and portends a poor prognosis [49]. We found significantly higher *EGFR* expression in the UMSCC-104 cell line, which originated from an HPV-positive tumor that did not respond to treatment [50], than in UMSCC-1 cells.

In addition to viral integration, DNA methylation can promote HPV-associated carcinogenesis by impairing E2 function, thereby increasing *E6* and *E7* oncogene expression [51, 52]. Methylation of the HPV LCR and L1 sequences has been associated with an increased grade of cervical neoplasia [8, 9], and LCR methylation is a known epigenetic mechanism [43, 53–56]. We found important differences in methylation in the cancer cell lines, with UMSCC-47 cells exhibiting hypermethylation (similar to CaSki cells), and UMSCC-104 cells displaying hypomethylation (similar to SiHa cells). The LCR methylation profiles obtained in this study could be used to differentiate the HNSCC cell lines by methylation status (hyper- or hypomethylated) (Table 3). However, an association between LCR methylation and cancer type was not observed. Instead, the LCR methylation pattern and integration status differed between the HNSCC cell lines: UMSCC-47 cells, with only integrated DNA (similar to SiHa cells), were hypermethylated (resembling CaSki cells), while UMSCC-104 cells, which contained mixed forms of HPV DNA, were hypomethylated, in agreement with the findings of others [45]. Thus, there was no observable relationship between the methylation pattern and the presence of integration.

However, in the HNSCC cell lines with predominantly integrated HPV, the methylation patterns may have been associated with the *E6* and *E7* expression profiles. *E7* expression was substantially greater in UMSCC-104 cells, which contained a hypomethylated LCR (0%), than in UMSCC-47 cells, which contained a hypermethylated LCR (76.9%). However, *E6* expression was only slightly higher in UMSCC-47 cells than in UMSCC-104 cells. These results suggest that LCR methylation may be more strongly associated with *E7* expression than with *E6* expression when HPV DNA is integrated.

To determine the association between *E7* expression and LCR methylation, we focused on the epigenetic regulation of oncogene transcription during HPV pathogenesis [10, 11]. The complete suppression of *E7* via hypermethylation of the LCR in UMSCC-47 cells clearly demonstrated this effect. Because most of the CpG sites in E2BS2 sequences were methylated in CaSki cells, we hypothesized that methylation may inhibit the binding of E2 to E2BS2, thus increasing *E6* and *E7* expression despite the presence of E2. However, when E2 is absent, as it is in SiHa and UMSCC-47 cells, LCR transcriptional regulatory sequences are the main target of methylation, leading to the suppression of *E7*. The high *E7* expression in UMSCC-104 cells with an unmethylated LCR and little to no E2 decisively indicated that methylation of the viral LCR strongly downregulates *E7* expression.

The few studies that have examined viral DNA methylation in HNSCC have reported divergent results [37, 38, 45, 57]. Wilson *et al*. [37] and Park *et al*. [38] reported hypomethylation of the LCR region in 3 and 22 OPC cases, respectively. Balderas-Loaeza *et al*. [57] reported hypermethylation in the LCR and an additional site in the *L1* gene in 10/12 oral carcinomas (83.3%). The promoter region was completely methylated in three of these cases, with an overall methylation rate of 28.2%. Reuschenbach *et al*. [45] assessed the methylation and viral integration status of 57 cases of OPC, and concluded that methylation of the LCR varied based on integration status. Tumors with complete methylation (greater than 80%) or low methylation (0–20%) were associated with integration, while those with an intermediate methylation pattern (20–80%) had predominantly episomal viral genomes.

Our analysis of clinical hgOED and HNSCC samples (Table 3) supported the previously documented conclusion that most malignant specimens are unmethylated, and that there are no significant methylation differences between premalignant and malignant lesions. Each of the three malignant HNSCC samples that were methylated had near-complete integration, with *E2/E6* ratios of 0.003 (HNSCC 7), 0.02 (HNSCC 2) and 0.03 (HNSCC 10). Methylated CpGs were located at various sites within the LCR, but no specific pattern was identified. Accordingly, while an increase in methylation may occur in some tumors, it does not appear to be a significant factor in most cases of HNSCC. While Balderas-Loaeza *et al*. reported hypermethylation in OSCC [57], we did not observe any LCR methylation in our cases of hgOED; thus, the involvement of methylation in oral premalignancy and OSCC remains unresolved.

The viral loads and integration rates of our hgOED samples suggested a potentially important relationship between histologic appearance and viral integration. As cytologic alterations increased across the three hgOED groups, the viral load and integration of HPV also increased (Table 2, Figure 3), with integration of HPV in all group 3 cases. In contrast, episomal HPV was the predominant form in group 1 hgOED cases. Thus, when increased cytologic changes (≥5.3/hpf) are observed during routine microscopic examination in a case of hgOED, there is a significant likelihood that HPV16 is present [3] and integrated into the host genome. It should be noted that only 25% of our malignant cases of OPC had episomal forms, while much higher rates have been reported in other studies [22, 32]. Carcinogenesis may still occur in cases with episomal DNA; indeed, this has been reported for the cervix, where episomal HPV still regulates oncogene activity [58], but may activate alternative oncogenic pathways [56].

Our finding of increasing integration with increasing cytologic alterations paralleled the pattern found in cervical cancer and was strikingly similar to the integration rates in our HNSCC cases and cell lines [7, 13–18]. Despite the inconclusive results of published work, we suspected that integration would be a significant factor in HNSCC, partially based on its high frequency of detection [24, 28, 59]. What is the clinical significance of the correlation we detected between viral integration and cytologic alterations? Could this histologic change herald malignant transformation? An expanded clinical study is needed to confirm the ability of our HPV-OED grading system to predict malignant transformation.

While the function of HPV as the driving force behind the malignant progression from hgOED to OSCC may be different than what has been established in OPC, it is reasonable to assume that many of the same mechanisms are involved. Accordingly, further analysis of HPV-OED cases may reveal HPV-induced transformative mechanisms that apply to all HPV-associated cases of HNSCC. Likewise, we believe that our examination of the viral integration and methylation of HNSCC cell lines can be effectively applied to HPV-associated cancers of the oral cavity.

## MATERIALS AND METHODS

### Sample collection

This study was approved by the Institutional Review Board at the University of Louisville. Formalin-fixed paraffin-embedded (FFPE) specimens of hgOED (*N* = 38) were obtained from the University of Louisville Oral Pathology Laboratory (Louisville, KY, USA) from April 2003 to February 2015 as previously described [3]. Fresh, frozen or FFPE tissues from cases of HNSCC (*N* = 50) were collected from 2006 to 2015 from the Cancer Database and Specimen Repository at the James Graham Brown Cancer Center (Louisville, KY, USA). University of Michigan Squamous Cell Carcinoma (UMSCC) oral cavity cancer cell lines (HPV-negative [UMSCC-1]; HPV-positive [UMSCC-47 and UMSCC-104]) [20, 50, 60] were purchased from EMD Millipore Corporation (Temecula, CA, USA) and cultured by standard protocols. Cervical cancer cell lines (CaSki and SiHa) were cultured as suggested by the American Type Culture Collection (Manassas, VA, USA).

### DNA extraction and HPV detection

**Figure 4:**
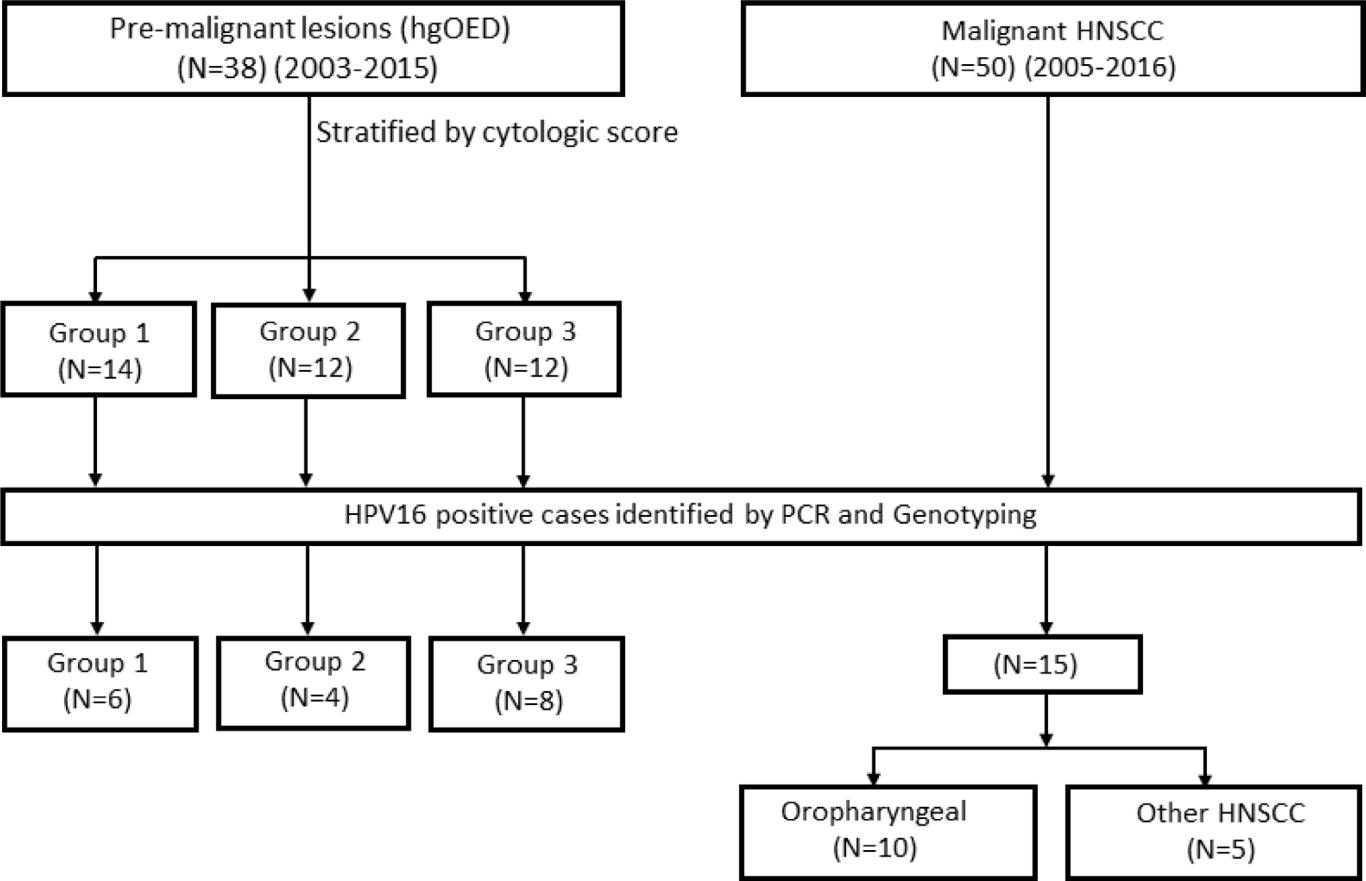
A flow diagram of premalignant and malignant case selection

DNA was extracted from fresh, frozen or FFPE specimens with a DNAeasy Blood & Tissue Kit (Qiagen, Germantown, MD, USA) per the manufacturer's instructions with RNase treatment (20 μL of 20 mg/mL). FFPE samples were deparaffinized with xylene and washed before DNA extraction. HPV detection and genotyping were performed with *16E7* primers (Supplementary Table 1), and *β-globin* was used as an internal control, as previously described [61]. HPV16-positive cases (18/38 for hgOED and 16/50 for HNSCC) were then used in HPV DNA integration and methylation studies. The method whereby cases were screened and selected for integration and methylation analyses is presented in Figure 4. Of the HNSCC cases, 10 were oropharyngeal and 5 were from other head and neck sites (2 oral cavity, 2 laryngeal and 1 hypopharyngeal).

### HPV integration and viral load assessment by quantitative PCR

We employed a previously described quantitative real-time PCR assay to evaluate the viral load and integration in cell lines and HNSCC specimens [44]. *E6* primers and probes were used to analyze the viral load in the cell, and *β-globin* was used as an internal control (Supplementary Table 1). We analyzed the copy numbers of target DNA per 20 ng of total genomic DNA using a quantitative PCR-based absolute quantification method [44]. In a total volume of 20 *μ*L, the final primer and probe concentrations and DNA template amounts were 0.3 μM, 0.1 μM and 20 ng, respectively. TaqMan™ Universal Master Mix II with Uracil-N-glycosylase was used according to the manufacturer's instructions (Thermo Fisher Scientific, Waltham, MA, USA). An HPV16 plasmid cloned in a pBR322 vector was used to plot a standard curve (300 pg to 0.3 pg). Water controls were included in each run. All experiments were performed in duplicate at least three times. We estimated the relative viral load by calculating the copy number ratio of *E6* in the specimen to *E6* in the SiHa cell line, which is known to contain two copies of HPV16 DNA per cell [44].

HPV integration status was evaluated based on the *E2/E6* ratio. The primers and probes were designed for specific amplification of the E2 hinge regions that are known to be disrupted most frequently during the process of viral integration (Supplementary Table 1) [44]. An *E2/E6* ratio ≥1 indicates episomal HPV DNA with no integrated forms, while a ratio of 0 indicates integrated HPV DNA with no episomal forms, and a ratio >0 and <1 indicates a “mixed” result of both episomal and integrated forms. Cervical cancer cell lines (CaSki and SiHa) were used as positive controls for the study of HPV DNA integration. We analyzed the copy numbers of HPV DNA per 20 ng of total genomic DNA in human tissue specimens by the same method. SiHa cells were also used as a reference to estimate the relative viral copy numbers of the tissue specimens.

### Determination of *E2* gene integrity

The integrity of the *E2* gene was determined by amplification of the full-length *E2* open reading frame (nucleotides 2755 to 3852 of NC_001526.2; primers 16E2a and E2b listed in Supplementary Table 1). Disruption of this region was defined as the absence of the full-length *E2* amplicon in agarose gel electrophoresis and a positive signal of parallel β-actin amplification. As CaSki cells carry an intact *E2* gene, the positive amplification signal of this ~1-kb full-length *E2* amplicon was used as a control. Amplification of the first half of *E2* (primers 16E2a and 16E2c) and the last half of the *E2* region (primers 16E2d and 16E2b) was performed for further confirmation of *E2* integrity.

### DNA methylation analysis: bisulfite sequencing

The methylation frequency of HPV DNA was profiled in each cancer cell line and in the cases of hgOED and HNSCC. Methylation on LCR sequences of HPV was assessed because this regulatory region is a known target of DNA methylation that induces transcriptional changes leading to cervical cancer [8, 9]. Purified genomic DNA was bisulfite-converted by means of an EpiTect^®^ Plus Bisulfite Kit (Qiagen, USA) according to the manufacturer's protocol. During this treatment, unmethylated cytosine residues are converted to uracil, whereas 5-methylcytosine is unaffected. Target DNA is then amplified by PCR, such that uracil residues are converted to thymine. The DNA methylation status of HPV DNA could thus be evaluated by the direct sequencing of the PCR products.

Because HPV16 has different variants (based on single-nucleotide polymorphisms present in the LCR and/or *E6*), an Asian-American variant of HPV16 (GenBank accession number: AF402678.1) was used as a reference HPV16 sequence for primer design and further analysis. The bisulfite-treated DNA was amplified by PCR with different sets of primers designed for the 15 CpG sites in the LCR region of HPV (Supplementary Table 1). Primers were designed within the consensus sequences among different variants of HPV16 (GenBank accession numbers: AF402678.1, AF125673.1, AY686584.1, NC_001526.2 and KF954093.1). Secondary primer sets were adopted in the event that PCR amplification with the primary primers was negative. As an internal control for the presence of bisulfite-modified DNA, we used primers that are specific to a modified region of the *β-actin* (*ACTB*) gene that lacks CpG sites. The PCR reaction mixtures consisted of 10X HiFi PCR buffer, 50 mM MgSO_4_, 10 mM deoxynucleotide triphosphates, 20 μM of each primer and 1 U of HiFi Taq polymerase (Thermo Fisher Scientific, USA) in a total volume of 20 μL. The PCR conditions were 2 min at 95° C; 45 cycles of 45 sec at 95° C, 45 sec at 52° C and 45 sec at 68° C; and 10 min at 68° C. The PCR products were separated on a 3% agarose gel, extracted with a QIAquick Gel Extraction Kit (Qiagen, USA) and sent to the DNA Core Facility at the University of Louisville for sequencing. Amplified products were directly sequenced with the same primers. Sequencing data were aligned with the NCBI BLAST database and the SeqMan Pro program (Lasergene 12, DNAstar Inc., Madison, WI, USA).

### RNA extraction and qRT-PCR analysis

Total RNA was isolated from cultured UMSCC-1, -47 and -104 cells with a PureLink^®^ RNA Mini Kit (Thermo Fisher Scientific, USA) and treated with DNase I, according to the manufacturer's instructions. Single-stranded complementary DNA (cDNA) was synthesized from 1 μg of total RNA with a SuperScript^®^ VILO cDNA Synthesis Kit (Thermo Fisher Scientific, USA) according to the manufacturer's instructions. qRT-PCR was performed in separate 20-μL reaction volumes to evaluate the expression of HPV16 genes *E6*, *E7* and *E2* and cellular genes *p16 (CDKN2a/INK4a)*, *EGFR* and *β-actin.* For the analysis of *E2* gene expression, primers were designed near the 5′ end and upstream of the frequent *E2* breakpoint to monitor the relative expression of truncated *E2* mRNA. qRT-PCR was performed in triplicate on an Applied Biosystems VIIa^TM^ 7 Real-Time PCR detection system (Thermo Fisher Scientific, USA) with 100 ng of cDNA as a template, along with the gene-specific forward and reverse primers (0.3 μM each) (Supplementary Table 1) and the Power SYBR^®^ Green Supermix (Thermo Fisher Scientific, USA). The amplification program for all primer sets was 95° C for 3 minutes, followed by 40 cycles of 95° C for 15 seconds and 60° C for 60 seconds. Real-time PCR amplification data were analyzed and cycle threshold (Ct) numbers were automatically determined by VIIa^TM^ 7 software v1.2.4 (Thermo Fisher Scientific, USA). The relative expression of each mRNA was calculated by the ΔCt method [62]. Endogenous *β-actin* mRNA levels were used for the normalization of mRNA expression. Due to the small amounts of mRNA recovered from clinical biopsies, qRT-PCR was only performed on cell lines and not on clinical biopsy samples.

### Statistical analysis

As the distribution of the viral load and the *E2/E6* ratio significantly departed from approximate normality, non-parametric tests were used to compare the distribution of these measures across the study groups. For both measures, the sample medians are reported, along with the first and third quartiles (representing the 25th and 75th percentiles, respectively). For pairwise comparisons, Wilcoxon rank-sum tests were conducted, and multiplicity-adjusted *p*-values (determined by Holm's method [63] to preserve the familywise error rate at less than or equal to 0.05) are reported. Given that the evaluated oropharyngeal cases (*N* = 10) could have exhibited different characteristics than HNSCC cases at other head and neck locations, a sensitivity analysis was conducted to assess only this group without the remaining non-oropharyngeal cases (*N* = 5). Statistical analyses were performed with the following functions from the R environment for statistical computing (version 3.4.2): *stats*, *ggplot2* and *ggsignif*. The relative gene levels in UMSCC cell lines were analyzed with GraphPad Prism 7 (GraphPad Software, La Jolla, CA, USA).

## SUPPLEMENTARY MATERIALS TABLES





## Abbreviations

hPV16: human papillomavirus type 16
HNSCC: head and neck squamous cell carcinoma
hgOED: high-grade oral epithelial dysplasia
HPV-OED: HPV-associated oral epithelial dysplasia
OSCC: oral squamous cell carcinoma
OPC: oropharyngeal squamous cell carcinoma
HRHPV: high-risk human papillomavirus
hpf: high-power field
PCR: polymerase chain reaction
E2BS: E2-binding site
LCR: long control region
qRT-PCR: quantitative reverse-transcription polymerase chain reaction
FFPE: formalin-fixed paraffin-embedded
UMSCC: University of Michigan Squamous Cell Carcinoma
cDNA: complementary DNA.
