# Supplementary material for: Viral DNA integration and methylation of human papillomavirus type 16 in high-grade oral epithelial dysplasia and head and neck squamous cell carcinoma
Source: Oncotarget. 2018 Jul 13;9(54):30419–33. doi: 10.18632/oncotarget.25754 (PMC6084396; doi:10.18632/oncotarget.25754)
Supplement: Supplementary file 1 [file oncotarget-09-30419-s001.pdf]

Viral DNA integration and methylation of human papillomavirus type 16 in high-grade oral epithelial dysplasia and head and neck squamous cell carcinoma

SUPPLEMENTARY MATERIALS

Supplementary Table 1: List of primers employed to detect HPV DNA, HPV integration, E2 gene integrity, bisulfite sequencing and qRT-PCR. See Supplementary_Table_1
