# Supplementary material for: Viral DNA integration and methylation of human papillomavirus type 16 in high-grade oral epithelial dysplasia and head and neck squamous cell carcinoma
Source: Oncotarget. 2018 Jul 13;9(54):30419–33. doi: 10.18632/oncotarget.25754 (PMC6084396; doi:10.18632/oncotarget.25754)
Supplement: Supplementary file 2 [file oncotarget-09-30419-s002.docx]

**Supplementary Table 1:** List of primers employed to detect HPV DNA, HPV integration, E2 gene integrity, bisulfite sequencing and qRT-PCR.

| **Target** | | **Primer** | **Sequence (5’-3’)** |
| --- | --- | --- | --- |
| **HPV detection** | | |  |
|  | L1 | MY09^*^ (For) | CGTCCMARRGGAWACTGATC |
|  |  | MY11^*^ (Rev) | GCMCAGGGWCATAAYAATGG |
|  | L1 | GP5+ (For) | TTTGTTACTGTGGTAGATACTAC |
|  |  | GP6+ (Rev) | GAA AAATAA ACTGTAAATCATATTC |
|  | β-globin | GH20 (For) | GAAGAGCCAAGGACAGGTAC |
|  |  | PC04 (Rev) | CAACTTCATCCACGTTCACC |
| **HPV16 detection** | | |  |
|  | 16E7 | For | TGAGCAATTAAATGACAGCTCAGAG |
|  |  | Rev | TGAGAACAGATGGGGCACACAAT |
| **HPV integration by qPCR** | | |  |
|  | E2 | 16E2F | AACGAAGTATCCTCTCCTGAAATTATTAG |
|  |  | 16E2R | CCAAGGCGACGGCTTTG |
|  |  | 16E2probe | (FAM)-CACCCCGCCGCGACCCATA-(TAMRA) |
|  | E6 | 16E6F | GAGAACTGCAATGTTTCAGGACC |
|  |  | 16E6R | TGTATAGTTGTTTGCAGCTCTGTGC |
|  |  | 16E6probe | (FAM)-CAGGAGCGACCCAGAAAGTTACCACAGTT-(TAMRA) |
| **E2 gene Integrity** | | |  |
|  | E2 (full length) | 16E2a (For) | ATGGAGACTCTTTGCCAACGTT |
|  |  | 16E2b (Rev) | TCATATAGACATAAATCCAGTAGAC |
|  | E2 (first half) | 16E2a (For) | ATGGAGACTCTTTGCCAACGTT |
|  |  | 16E2c (Rev) | TTATTCTTTGATACAGCCAGTGTTG |
|  | E2 (last half) | 16E2d (For) | CCTCACTGCATTTAACAGCTCA |
|  |  | 16E2b (Rev) | TCATATAGACATAAATCCAGTAGAC |
| **Bisulfite sequencing** | | |  |
|  | 5’-LCR and enhancer | (2F+3R) For | GTGTATGTGTTTTTAAATGTTTGTGT |
|  |  | (2F+3R) Rev | CACAATATACATAATAATTCAATAATTAC |
|  | enhancer | (4F+4R) For | GTAATTATTGAATTATTATGTATATTGTG |
|  |  | (4F+4R) Rev | CACACACCCATATACAATTTTACAA |
|  | promoter | (5F+5R)For | TTGTAAAATTGTATATGGGTGTGTG |
|  |  | (5F+5R) Rev | ACAACTCTATACATAACTATAATAACT |
|  | 5’-LCR and | BSP-6 (For) | TAAATTATATTTGTTATATTTTGTTTTTGT |
|  | enhancer | BSP-6 (Rev) | TAATTAACCTTAAAAATTTAAACCTTATAC |
|  | β-actin | mACTB (For) | TGGTGATGGAGGAGGTTTAGTAAGT |
|  |  | mACTB (Rev) | AACCAATAAAACCTACTCCTCCCTTAA |
| **Gene expression by qRT-PCR** | | |  |
|  | E6 | For | CAGCAATACAACAAACCG |
|  |  | Rev | GCAACAAGACATACATCG |
|  | E7 | For | CAGAGGAGGAGGATGAAATAG |
|  |  | Rev | AGGTCTTCCAAAGTACGAATG |
|  | E2 | For | TGATAGTACAGACCTACGTGACCATATAGA |
|  |  | Rev | CCCATTTCTCTGGCCTTGTAAT |
|  | p16^INK4a^ | For | CATAGATGCCGCGGAAGGT |
|  |  | Rev | CCCGAGGTTTCTCAGAGCCT |
|  | EGFR | For | GGAGAACTGCCAGAAACTGACC |
|  |  | Rev | GCCTGCAGCACACTGGTTG |
|  | β-actin | For | CCATCGTCCACCGCAAAT |
|  |  | Rev | GCTGTCACCTTCACCGTTCC |
|  | IPO8 | For | CAGTGCATTCCACTCTTCGT |
|  |  | Rev | ACGAAGCTCACTAGTTTTGACC |
